# ADULT INFECTIONS ARE ASSOCIATED WITH HIGHER RISK OF ALZHEIMER’S DISEASE BUT LOWER RISK OF CANCER

**DOI:** 10.1093/geroni/igac059.2775

**Published:** 2022-12-20

**Authors:** Svetlana Ukraintseva, Vladimir Popov, Hongzhe Duan, Arseniy Yashkin, Igor Akushevich, Konstantin Arbeev, Anatoliy Yashin

**Affiliations:** Duke University, Durham, North Carolina, United States; Duke University, Durham, North Carolina, United States; Duke University, Durham, North Carolina, United States; Duke University, Durham, North Carolina, United States; Duke University, Durham, North Carolina, United States; Duke University, Durham, North Carolina, United States; Duke University, Durham, North Carolina, United States

## Abstract

Growing evidence suggests that infections may promote Alzheimer’s disease (AD). Chronic infections have also been linked to several cancers. However, there is evidence that acute febrile infections may treat some cancers, suggesting that anti-pathogen immune response could help fight off transformed cells. To explore potentially ambivalent role of infections in cancer and AD, we estimated associations between history of infection and subsequent risks of AD, cancer (all sites combined), and all-cause mortality, in sample of 270K participants of the UK Biobank. We found that prior infection is associated with 29% higher risk of AD (RR=1.29, 95% CI[1.02,1.63], both sexes). In contrast, the overall cancer risk was 49% lower in people with history of infection, compared to individuals without such history (RR=0.49, 95% CI[0.46,0.52], both sexes). History of infections was also associated with 7% reduced total survival at ages 70+ (RR=0.93, 95% CI[0.92,0.94]); however, the negative effect was less evident at younger ages. Results of our study suggest that infections may antagonistically influence chances of AD and cancer. They also show that impact of infections on survival depends on age and is more detrimental at older ages, which may reflect general decline in immune resilience due to aging, as well as the fact that cancer becomes a major contributor to mortality risk at younger ages than AD. Understanding the trade-offs between major diseases is essential for optimizing disease prevention and pro-longevity interventions, since measures aiming to prevent one disease may sometimes increase risks of other major conditions and/or all-cause mortality.

